# Transcriptomic and Metabolomic Analyses Reveal the Phenylpropanoid Metabolism Regulatory Network in *Arabidopsis* Response to *Colletotrichum higginsianum* Infection

**DOI:** 10.3390/life16091505

**Published:** 2026-09-09

**Authors:** Hong Ye, Qiwen Gao, Yongjian Zou, Chengnv Liao, Bi-Er Lai, Yingying Li, Mingyang Din, Chang Liu, Xiuhua Gu, Jiayi Jiang, Zhuoyun You, Yukun Wang

**Affiliations:** 1 Guangdong Provincial Key Laboratory of Utilization and Conservation of Food and Medicinal Resources in Northern Region; College of Biology and Agriculture, Shaoguan University, Shaoguan 512005, China; 19881212hong@163.com (H.Y.); 19147909737@163.com (Q.G.); z1962678600@163.com (Y.Z.); 15992998135@163.com (C.L.); 13510508968@163.com (B.-E.L.); 13533582280@163.com (Y.L.); 13417260602@163.com (X.G.); 18898849991@163.com (J.J.); 17825698867@163.com (Z.Y.); 2 Guangdong Provincial Engineering and Technology Research Center of Special Fruit and Vegetables in Northern Region; Engineering and Technology Research Center of Shaoguan Horticulture in Shaoguan University, Shaoguan University, Shaoguan 512005, China; 3 College of Life Sciences, South China Agricultural University, Guangzhou 510642, China; dingmingyang@stu.scau.edu.cn; 4 College of Life Sciences, Shanghai Normal University, Shanghai 200234, China; 13537035862@163.com

**Keywords:** *Colletotrichum higginsianum*, transcriptome, metabolome, fungi infection, pathogen infection, *Arabidopsis*

## Abstract

*Colletotrichum higginsianum* (*Ch*) is a typical hemibiotrophic ascomycetous fungus. The diseases it causes often lead to considerable economic losses in global cruciferous crop production. However, current knowledge is still insufficient for us to gain a deeper understanding of how host plants respond at the transcriptional level during *Ch* infection. Herein, we performed transcriptomic and metabolic assays between Mock and *Ch*- infected samples. The results showed that *Ch* infection significantly inhibited the shoot fresh weight and primary root length of host plants. Furthermore, gene ontology (GO) terms and Kyoto Encyclopedia of Genes and Genomes (KEGG) pathways related to phenylpropanoid metabolism were highly enriched. Defense hormone salicylic acid (SA) and three metabolites belonging to the phenylpropanoid metabolism pathway were identified. Meanwhile, we screened 5 related enzyme-encoding genes and 78 transcription factors (TFs). Five WRKY, three MYB, and two NAC TFs showed significant expression changes and high correlation with enzyme genes. Our results enrich regulatory networks in crucifier pathogen responses. This work provides potential molecular candidates to support subsequent studies on the mechanisms underlying *Ch* resistance.

## 1. Introduction

*Colletotrichum*, a core group of filamentous fungal plant pathogens, exhibits high taxonomic diversity and strong pathogenic relevance. Its taxonomic system has been continuously refined with the advancement of research techniques: early studies suggested that it comprises 9 core clades, 15 major species complexes, and 252 species [1]. However, with the application of multilocus sequence analysis combined with molecular techniques and phylogenomics, the genus has been further classified into 280 species, which are grouped into 16 main species complexes and 15 singleton species [2,3,4,5,6]. In terms of pathogenic characteristics, species within this genus employ diverse strategies to invade host tissues, ranging from intracellular hemibiotrophy to subcuticular intramural necrotrophy [7], and cause anthracnose spots and blights in economically important crops such as fruits, vegetables, and ornamentals [8]. Specifically, *C. siamense* (belonging to the *C. gloeosporioides* complex) is a dominant pathogen in field-grown tropical crops (e.g., rubber trees, mangoes, and lychees) [9,10,11]. For species identification of *Colletotrichum*, traditional methods rely on professional experience owing to the morphological similarities among its species [12]. The development of whole-genome sequencing technology has provided critical support for clarifying the genus’ evolutionary origin and analyzing its species complexes. In scientific research, *Colletotrichum* has long served as a model pathogen for basic mechanistic research [8].

*Ch* is a prime example of a hemibiotrophic ascomycetous fungus that causes anthracnose on a wide range of cruciferous plants, including economically important *Brassica* crops and the model plant *Arabidopsis* [13]. The infection process of *Ch* constitutes a highly regulated sequence of developmental events. It begins with the adhesion of a conidium to the host surface, followed by germination and differentiation of a specialized infection structure called an appressorium. This melanized, dome-shaped cell generates immense turgor pressure to breach the host cuticle and cell wall via a narrow penetration peg [14]. Once inside an epidermal cell, the fungus develops large, bulbous biotrophic hyphae that are separated from the host cytoplasm by an interfacial membrane. During this biotrophic phase, the fungus secretes a battery of effector proteins thought to manipulate host cellular processes and suppress defense responses [15,16]. After a period of biotrophy, the fungus switches to necrotrophy, forming thin, filamentous secondary hyphae that ramify throughout the host tissue and cause extensive cell death and lesion development [17]. The genetic tractability of both *Ch* and its host *Arabidopsis* has established this pathosystem as an invaluable model for dissecting the molecular basis of hemibiotrophic interactions [13].

In response to pathogen attack, plants deploy a multi-layered innate immune system consisting of PAMP-triggered immunity (PTI) and effector-triggered immunity (ETI). PTI is activated when cell-surface pattern recognition receptors perceive conserved pathogen-associated molecular patterns such as fungal chitin, initiating downstream signaling cascades including ion fluxes, reactive oxygen species (ROS) bursts, and mitogen-activated protein kinase signaling to facilitate cell wall reinforcement and transcriptional reprogramming of defense genes [15]. As a counter-defense, pathogens secrete effector proteins to suppress PTI; plants subsequently activate ETI, wherein intracellular nucleotide-binding leucine-rich repeat proteins recognize pathogen effectors and often elicit robust immune responses accompanied by hypersensitive programmed cell death to restrict pathogen proliferation. These immune outputs are coordinately modulated by phytohormones, primarily salicylic acid (SA), jasmonic acid (JA), and ethylene (ET). Generally, SA signaling mediates resistance to biotrophic and hemibiotrophic pathogens, whereas JA/ET-dependent pathways dominate defenses against necrotrophs. Downstream of such immune signaling lies the biosynthesis of diverse antimicrobial secondary metabolites, exemplified by the *Arabidopsis* phytoalexin camalexin [18]. The phenylpropanoid pathway represents an ancient, central secondary metabolic hub that converts shikimate pathway-derived phenylalanine into more than 800 distinct metabolites, with the deamination of phenylalanine to cinnamic acid catalyzed by phenylalanine ammonia-lyase (PAL) serving as the committed step [19,20,21]. Metabolites derived from this pathway contribute substantially to plant defense: lignin strengthens cell walls as a physical barrier against pathogen invasion, flavonoids act as antimicrobial phytoalexins or signaling mediators in plant–microbe interactions, and phenolic acids including sinapic acid mitigate pathogen-induced oxidative stress [21,22,23,24,25]. Notably, SA is biosynthesized via the phenylpropanoid pathway, establishing a direct molecular link between phenylpropanoid metabolism and phytohormone-regulated immune signaling networks [23].

The regulation of the phenylpropanoid pathway is largely controlled at the transcriptional level by various families of transcription factors (TFs) that bind to specific cis-regulatory elements in the promoters of biosynthetic genes. Among the most prominent regulators are members of the R2R3-MYB family of TFs, which act as master switches, both activating and repressing different branches of the pathway [26,27]. In addition to MYBs, TFs from the WRKY and NAC families are also known to be involved in regulating stress-induced phenylpropanoid biosynthesis, often forming complex regulatory networks that fine-tune the metabolic output in response to specific stimuli. Understanding how these TFs orchestrate the expression of phenylpropanoid genes is key to deciphering the plant’s defense strategy.

Despite the established importance of phenylpropanoid metabolism in plant defense, a comprehensive, systems-level understanding of how this pathway is transcriptionally and metabolically reprogrammed in crucifers during infection by the hemibiotroph *Ch* remains incomplete. Previous research has provided valuable insights by focusing on specific aspects of the interaction, such as the identification of individual fungal pathogenicity genes [28], the role of specific host defense components [29], or the response of other metabolic networks like indolic secondary metabolites [18]. While some transcriptomic analyses have been performed, they have often focused on specific aspects like small RNA dynamics [30] or comparative expression profiling [31]. However, a detailed integration of transcriptomic and metabolomic data to specifically map the regulation of the phenylpropanoid pathway during *Ch* infection is still needed to build a holistic view of the host response.

Herein, we employed a combined transcriptomic and metabolomic approach to investigate the global changes in host gene expression and metabolite accumulation in response to *Ch* infection. Our study aimed to (i) characterize the physiological and metabolic impact of the infection on the host plant, (ii) identify the key metabolic pathways, with a particular focus on phenylpropanoid metabolism, that are significantly altered, and (iii) screen for candidate enzyme-encoding genes and upstream TFs, such as WRKYs, MYBs, and NACs, that likely orchestrate these metabolic shifts. By integrating these multi-omics datasets, we sought to construct a more complete regulatory network underlying the host defense response. This work provides novel insights into the molecular mechanisms of crucifer resistance to anthracnose and identifies promising molecular candidates for future functional validation and potential application in breeding disease-resistant crops.

## 2. Materials and Methods

### 2.1. Plant Material and Growth Conditions

The *Arabidopsis* accession Col-0 was used as the wild type (WT). Seeds were surface sterilized with 6% sodium hypochlorite plus 0.1% Triton X-100 for 15 min and then rinsed with autoclaved distilled water 3 times. The seeds were sown on a half-strength Skoog–Murashige (MS) agar medium with 25 mM sucrose. After incubation at 4 °C for 2 days, the plates were placed vertically in a plant growth chamber under 16 h light/8 h dark at 22 °C.

### 2.2. Culture of Ch and Plant Infection

The fungal isolate was recovered from symptomatic radish grown in an open field at Shaoguan University and purified on PDA medium at 25 °C. Genomic DNA was extracted from fresh mycelium. Four loci (ITS, TUB2, ACT, and GAPDH) were amplified and sequenced following published protocols for the *Colletotrichum destructivum* species complex. Consensus sequences were aligned with MAFFT (v7.526), and a concatenated multilocus dataset was generated. Maximum-likelihood phylogenetic analysis was performed in IQ-TREE with 1000 bootstrap replicates, using published sequences of ex-type strains of taxa within the *C. destructivum* species complex as references. Combined with morphological features and host information, the isolate was identified as *Ch*. For fungal inoculation, 7 days-old *Arabidopsis* seedlings grown in half MS with 25 mM sucrose were transferred to phosphate (Pi)-sufficient (625 μM KH_2_PO_4_) half-strength MS medium without sucrose, and 9 days-old agar plugs of *Ch* hyphae, cultured on PDA medium, were inoculated at a distance of 3.2 cm from the initial position of the seedling roots, with 10 plant seedlings and 4 agar plugs of *Ch* hyphae per plate. For the control plants, the same methods and treatments were performed using agar plugs without *Ch*.

### 2.3. Observation of Physiological Indicators of Host

Shoot fresh weight and primary root length were determined at 14 dpi. In the present study, we specifically focused on the late infection stage at 14 dpi, which represents the critical symptomatic phase of leaf necrosis and severe disease occurrence in this pathosystem. The primary root length was measured using Fiji (ImageJ2). Individual seedlings were measured in these experiments.

### 2.4. Sample Collection

The 14 dpi seedlings and the Mock seedlings (whole seedlings) were collected using sterile tweezers in a laminar flow clean bench and frozen in liquid nitrogen immediately. For RNA-seq, at least 0.3 g tissues were collection for each sample; for widely targeted metabolome analysis, at least 0.5 g tissues were collection for each sample.

### 2.5. RNA-Seq and Data Analysis

The RNA Easy Fast Plant Tissue Kit (TIANGEN, Beijing, China) was used to extract total RNA. The RNA quality was monitored using a micro-spectrophotometer (NanoDrop 2000c, ThermoFisher, Waltham, MA, USA). Total RNA was quantified using a spectrophotometer (Thermo Fisher Scientific, Wilmington, DE, USA), and RNA integrity was assessed with an Agilent 2100 Bioanalyzer to obtain RIN values (mean RIN ≥ 7.0) as well. RNA libraries were constructed using the TruSeq Stranded mRNA Library Prep Kit and sequenced on the Illumina NovaSeq 6000 platform with paired-end 150 bp reads. Raw sequencing reads were trimmed to remove adapter sequences and low-quality reads using fastp software (v1.3.6). Clean reads were aligned to the *Arabidopsis* reference genome (TAIR10) using HISAT2. The methods of the RNA-seq data analysis were clearly described previously [32,33]. Genes with an adjusted *p*-value < 0.05 found by DESeq were assigned as differentially expressed. For primary screening, genes with a fold change ≥ 1 and false discovery rate < 0.05 were considered as DEGs. To further narrow down the number of DEGs, the thresholds for gene expression fold change were increased. The fragments per kilobase of transcript per million mapped (FPKM) reads method was used to calculate the expression abundances of the genes. Gene set enrichment analysis was performed against the all-annotated genes of *Arabidopsis* (TAIR10) as the background gene set. The GO enrichment analysis of DEGs was implemented using the GOseq R packages based on the Wallenius noncentral hypergeometric distribution. KEGG pathway analysis was performed (*p* ≤ 0.05) using BlastX (v2.15.0) searches against the KEGG pathway database (https://www.genome.jp/kegg/pathway.html, accessed on 20 March 2026). GO functional annotations were derived from TAIR10, and KEGG pathway enrichment was implemented based on KEGG Release 109. The experiment was conducted in 3 independent biological replicates.

### 2.6. Gene Validation

Reverse-transcription PCR was performed using the FastKing RT Kit (With gDNase) (TIANGEN, China). qRT-PCR was applied as described previously [32]. Each experiment was repeated three times. The relative expression level of each gene was calculated using the 2^−ΔΔCt^ method. The *Arabidopsis actin2* gene was used as the inner reference [33]. The primers are listed in Table S1.

### 2.7. Widely Targeted Metabolome Analysis

The method of widely targeted metabolome analysis was mainly referenced from the previous study [34]. Briefly, *Arabidopsis* seedlings were freeze-dried using a vacuum freeze-dryer. We dissolved 50 mg of the freeze-dried sample in 1.0 mL of solution (methanol:acetonitrile:H_2_O = 1:2:1) and vortexed 30 s. The above solution was crushed using a mixer mill with a bead for 10 min at 45 Hz and sonicated in an ice-water bath for 10 min. We placed the sample in a refrigerator at −20 °C for 1 h. Following centrifugation at 4 °C and 12,000 rpm for 15 min, the 300 uL supernatant was filtered (0.22 μm pore size) into a 2 mL injection bottle, and 10 μL of each sample were taken and mixed into QC samples before UPLC-MS/MS analysis. The sample extracts were analyzed using a UPLC-ESI-MS/MS system (UPLC, Waters Acquity I-Class PLUS; MS, Applied Biosystems QTRAP 6500+). The analytical conditions were as follows: UPLC column, Waters HSS-T3 (1.8 µm, 2.1 mm × 100 mm); the mobile phase consisted of solvent A, pure water with 0.1% formic acid and 5 mM Ammonium acetate, and solvent B, acetonitrile with 0.1% formic acid. Sample measurements were performed with a gradient program that employed starting conditions of 98% A and 2% B and was held for 1.5 min. Within 5.0 min, a linear gradient to 50% A, 50% B was programmed; within 9.0 min, a linear gradient to 2% A, 98% B was programmed, and a composition of 2% A, 98% B was kept for 1 min. Subsequently, a composition of 98% A, 2% B was adjusted within 1 min and kept for 3 min. The flow velocity was set to 0.35 mL per minute; the column oven was set to 50 °C; the injection volume was 4 ul. The effluent was alternatively connected to an ESI-triple quadrupole-linear ion trap (QTRAP)-MS. The ESI source operation parameters were as follows: source temperature 550 °C; ion spray voltage (IS) 5500 V (positive ion mode)/−4500 V (negative ion mode); ion source gas I, gas II, and curtain gas (CUR) were set at 50, 55, and 35 psi, respectively; the collision-activated dissociation (CAD) was medium. Instrument tuning and mass calibration were performed with 10 and 100 μmol/L polypropylene glycol solutions in QQQ and LIT modes, respectively. QQQ scans were acquired as MRM experiments with collision gas (nitrogen) set to medium. DP (declustering potential) and CE (collision energy) for individual MRM transitions were determined with further DP and CE optimization. A specific set of MRM transitions was monitored for each period according to the metabolites eluted within this period.

After normalizing the original peak area information with the total peak area, follow-up analysis was performed. Principal component analysis and Spearman correlation analysis were used to judge the repeatability of the samples within the group and the quality of control samples. According to the grouping information, the difference multiples were calculated and compared; a T-test was used to calculate the difference significance *p*-value of each compound. The R language package was used to perform OPLS-DA modeling, and the parameters were as follows: R2X = 0.72, R2Y = 0.94, and Q2 = 0.81. A permutation test (*n* = 200) showed that the intercept of Q2 was −0.13, indicating no overfitting of the model. The metabolomic detection panel contained 3000 predefined metabolites. The VIP value of the model was calculated using multiple cross-validation. The method of combining the difference multiple, the *p*-value, and the VIP value of the OPLS-DA model was adopted to screen the differential metabolites. The screening criteria were |FC| > 1, *p*-value < 0.05, and VIP > 1. For metabolites with signal intensity below the instrumental detection threshold (signal intensity = 10) in any group, a fixed value of 10 was imputed to facilitate fold change and log_2_FC calculation. This widely targeted metabolomic analysis was performed without external standard calibration for absolute quantification. Multiple-testing correction was not performed for the metabolomic dataset, given the limited total number of metabolite features detected (1500 features), which lowers the risk of false-positive inflation. A total of 92 DAMs were processed using this imputation strategy. The identified compounds were searched for regarding their classification and pathway information in the KEGG (http://www.kegg.jp/kegg/compound/, accessed on 10 March 2026), HMDB (https://hmdb.ca/metabolites/, accessed on 10 March 2026), and lipidmaps (https://lipidmaps.org/, accessed on 11 March 2026) databases. The significance in the differences in metabolites in KEGG pathway (http://www.kegg.jp/kegg/pathway.html, accessed on 11 March 2026) enrichment was calculated using the hypergeometric distribution test. The experiment was conducted using 3 independent biological replicates.

### 2.8. Correlation Network Analysis

Pairwise correlations between gene expression and metabolite abundance were computed by Pearson correlation analysis. Multiple testing correction was performed using the Benjamini–Hochberg algorithm to control the false discovery rate (FDR). Correlations with FDR < 0.05 and |r| > 0.8 were included for network construction. Nodes with the top 10% of node degree were identified as hub nodes in the correlation network.

### 2.9. Statistical Analysis and Data Availability

A Student’s *t*-test (two-tailed) was performed using SPSS software (v. 22) to detect differences. The RNA-seq data sets were submitted to the National Genomics Data Center (https://ngdc.cncb.ac.cn/, accessed on 20 March 2026) with the accession number CRA040369.

## 3. Results

### 3.1. Inoculation of Ch on Root Tissue Inhibited the Growth of Arabidopsis

**Figure 1:**
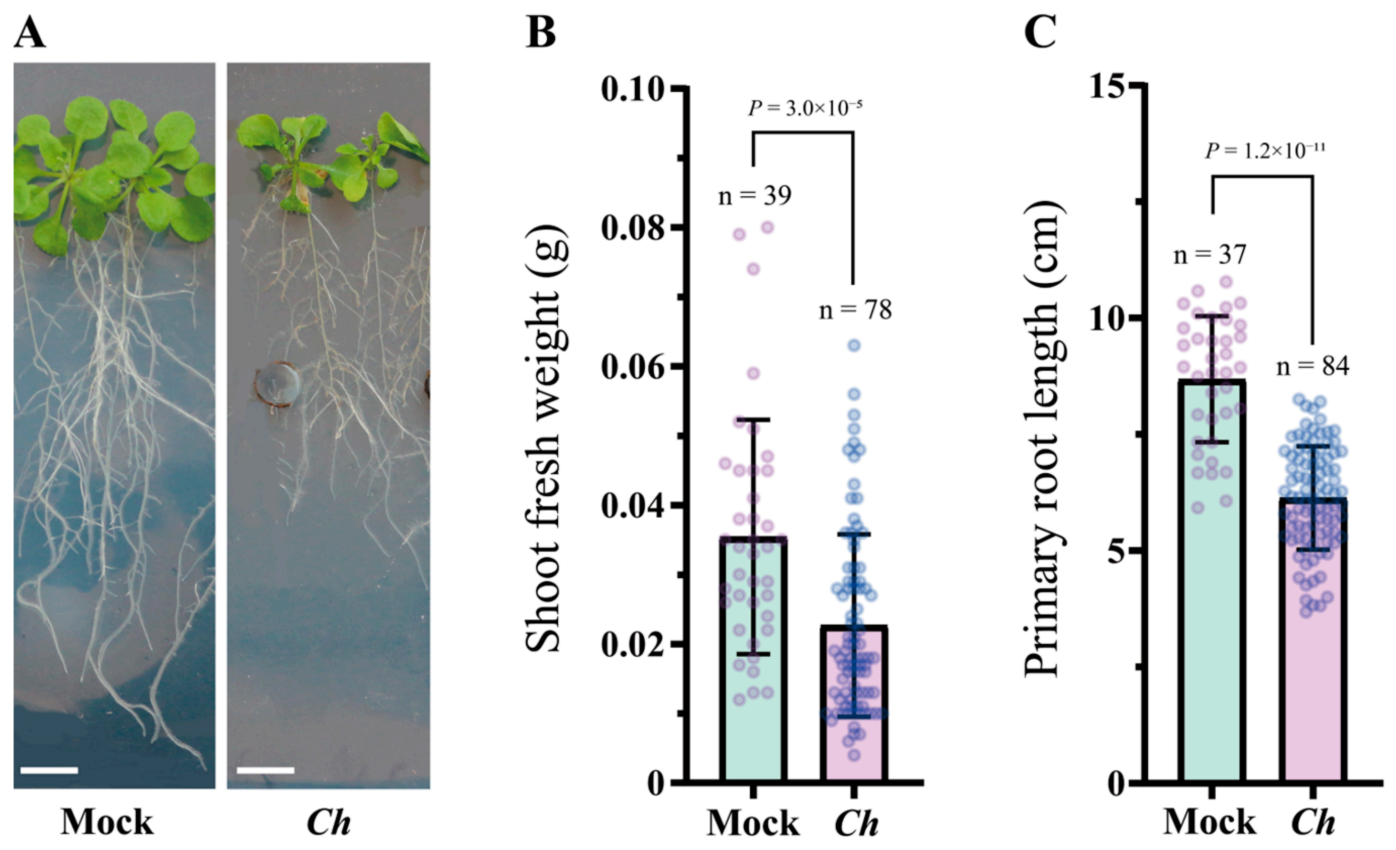
Observation of *Arabidopsis* seedlings after infection with *Ch*. (**A**) Phenotype of *Arabidopsis* seedlings after infection with *Ch*. Mock: control group; *Ch*: infected group. Scale bars = 1 cm; (**B**) shoot fresh weight of Mock and *Ch* groups. n: the number of observed seedlings. Unequal sample sizes were caused by differences in seed germination across plates. Student’s *t*-test was performed. (**C**) Primary root length of Mock and *Ch* groups. n: the number of observed seedlings. Unequal sample sizes were caused by differences in seed germination across plates. Student’s *t*-test was performed.

In order to understand the influence of *Ch* infection on the growth of *Arabidopsis*, we inoculated this fungus onto roots via gel blocks covered with hyphae. We first observed phenotypes of seedlings at 14 days post-inoculation (dpi). As shown in Figure 1A, there were clear differences between Mock (without inoculation) and *Ch* (with *Ch* infection) seedlings. The seedlings inoculated with *Ch* showed curled leaf edges and shorter roots than those of the Mock seedlings (Figure 1A). The quantitative data demonstrated that the shoot fresh weight of Mock seedlings was substantially higher than that of *Ch* seedlings (Figure 1B). In agreement with phenotypic observations, the length of primary roots of Mock seedlings was significantly longer than that of the *Ch*-infected seedlings (Figure 1C). These results indicated that *Ch* infection resulted in the growth inhibition of *Arabidopsis* seedlings.

### 3.2. Overview of the Transcriptome Data

**Figure 2:**
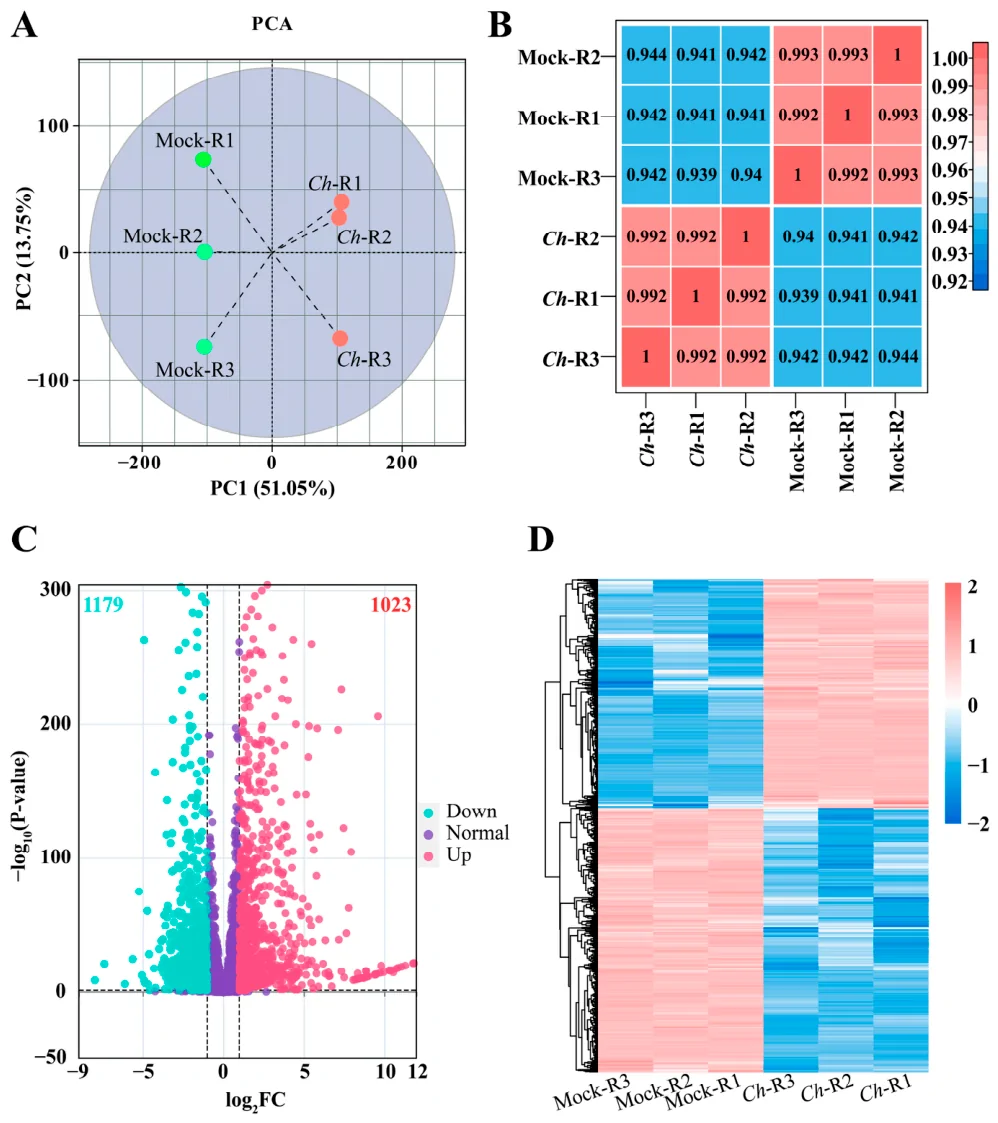
Overview of the transcriptomic data. (**A**) Principal component analysis of Mock and *Ch* groups; (**B**) duplicate relevance assessment of Mock and *Ch* groups; (**C**) volcano plot of DEGs. Upregulated and downregulated genes were marked in red and green, respectively; (**D**) heatmap of all DEGs. The FPKM value of each gene was normalized as the z-score value. Genes with similar expression profiles were clustered in the same group.

Given that *Ch* infection reduced the growth of *Arabidopsis* seedlings, we then performed transcriptome analysis using the Mock and *Ch* samples, which aimed to uncover candidate genes responsive to *Ch* infection. Total reads of each RNA-Seq library exceeded 41,048,144, and clean reads exceeded 20,524,072. The corresponding clean bases exceeded 6,132,557,135. Meanwhile, the GC content and Q30 ratio were higher than 44.89% and 96.87%, respectively. Moreover, more than 98.28% of total reads were mapped to the reference genome, and over 95.21% of mapped reads were uniquely mapped to the reference genome (Table S2), indicating that the RNA-Seq data were of high quality. The results of principal component analysis (PCA) showed that the variance explained by PC1 and PC2 was 51.05% and 13.75%, respectively. The two principal components together explained 64.8% of the variation in the original data, and the major variation originated from treatment groups, suggesting high reliability of the analysis (Figure 2A). Sample correlation analysis revealed that the correlation coefficient among samples within the same treatment was very high (>0.992), whereas the correlation between Mock and *Ch* samples was low (Figure 2B). Furthermore, we identified DEGs with a fold change ≥ 2 and FDR < 0.01, as we sought to narrow down the number of DEGs. A total of 2202 DEGs were screened, including 1023 upregulated and 1179 downregulated genes (Figure 2C). Expression pattern analysis revealed that all DEGs were clearly clustered into two distinct groups: one set was highly expressed in the Mock group but weakly expressed in *Ch*, while the other exhibited the opposite expression pattern, i.e., highly expressed in *Ch* but weakly expressed in Mock (Figure 2D).

### 3.3. Go Enrichment and Kegg Pathway Analysis of DEGs

**Figure 3:**
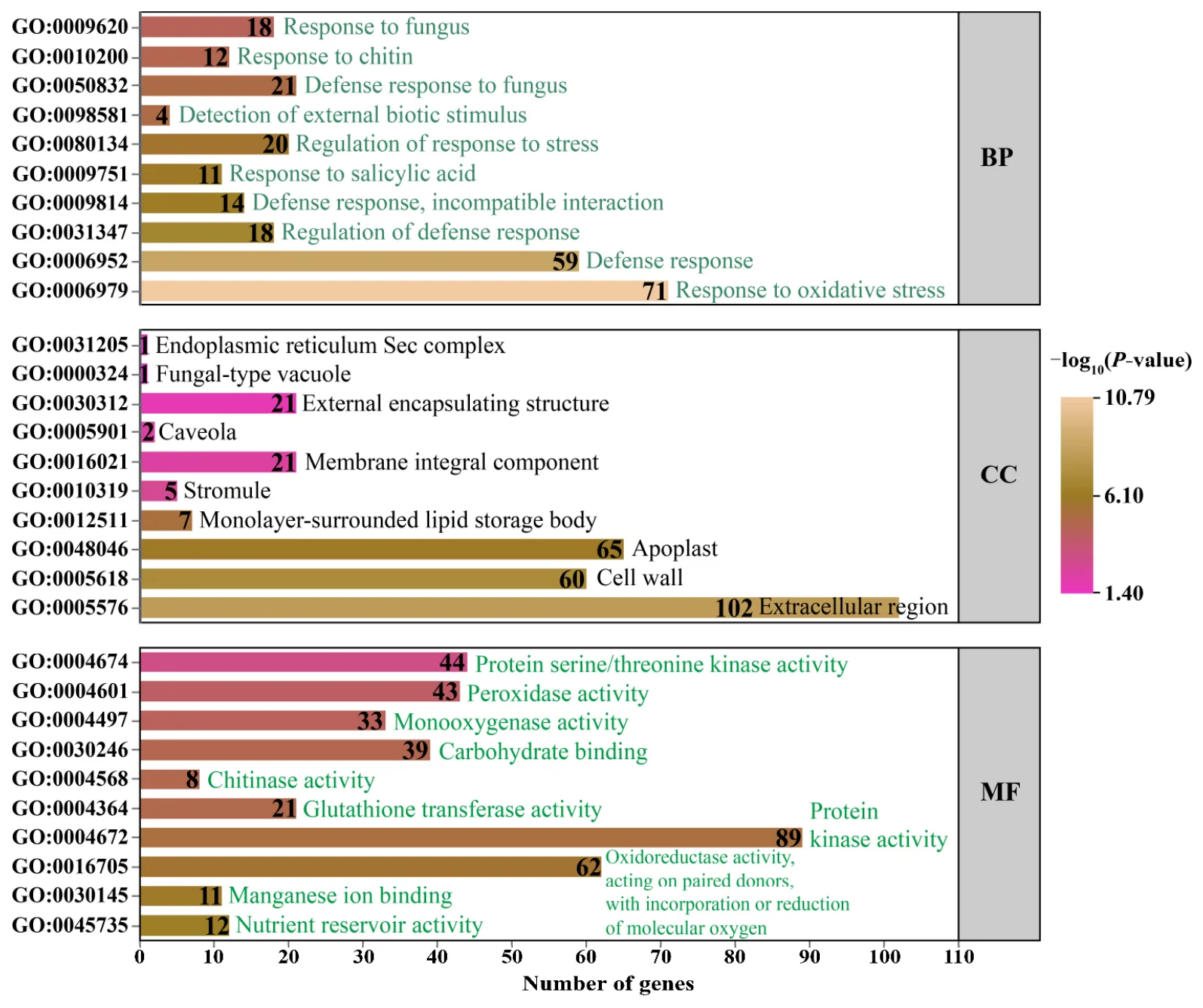
GO analysis of DEGs and the top 10 terms of each ontology were displayed. The number of DEGs in each GO term was marked in the column. BP: biological process; CC: cellular component; MF: molecular function.

To explore the potential functions of DEGs, GO enrichment analysis was performed. Based on the GO database, all DEGs were assigned to three main categories: molecular function (MF), cellular component (CC), and biological process (BP). As shown in Figure 3, the top 10 enriched GO terms for MF, CC, and BP are presented separately. Within the BP category, 248 DEGs were enriched in the top 10 GO terms. Specifically, 18 DEGs were associated with ‘Response to fungus’ (GO:0009620); 12 DEGs belonged to the term ‘Response to chitin’ (GO:0010200); 21 and 4 DEGs were identified in ‘Defense response to fungus’ (GO:0050832) and ‘Detection of external biotic stimulus’ (GO:0098581), respectively; the remaining 173 DEGs were enriched in ‘Response to salicylic acid (SA)’ (GO:0009751, 11 DEGs), ‘Defense response, incompatible interaction’ (GO:0009814, 14 DEGs), ‘Regulation of defense response’ (GO:0031347, 18 DEGs), ‘Defense response’ (GO:0006952, 59 DEGs), ‘Regulation of response to stress’ (GO:0080134, 20 DEGs), and ‘Response to oxidative stress’ (GO:0006979, 71 DEGs) (Figure 3).

For the CC category, 285 DEGs were enriched within the top 10 terms. A total of 65, 60, and 102 DEGs were associated with ‘Apoplast’ (GO:0048046), ‘Cell wall’ (GO:0005618), and ‘Extracellular region’ (GO:0005576); 21 and 21 DEGs were enriched in ‘External encapsulating structure’ (GO:0030312) and ‘integral component of membrane’ (GO:0016021), respectively. Furthermore, 16 DEGs were distributed across ‘Endoplasmic reticulum Sec complex’ (GO:0031205, 1 DEG), ‘Fungal-type vacuole’ (GO:0000324, 1 DEG), ‘Caveola’ (GO:0005901, 2 DEGs), ‘Stromule’ (GO:0010319, 5 DEGs), and ‘Monolayer-surrounded lipid storage body’ (GO:0012511, 7 DEGs) (Figure 3).

Moreover, 362 DEGs were enriched in the top 10 MF GO terms. Among these terms, ‘Protein kinase activity’ (GO:0004672) contained 89 DEGs and ‘Oxidoreductase activity, acting on paired donors, with the incorporation or reduction in molecular oxygen’ (GO:0016705) included 62 DEGs. In addition, 44, 43, 39, and 33 DEGs were enriched in ‘Protein serine/threonine kinase activity’ (GO:0004674), ‘Peroxidase activity’ (GO:0004601), ‘Carbohydrate binding’ (GO:0030246), and ‘Monooxygenase activity’ (GO:0004497), respectively. Finally, 21, 8, 12, and 11 DEGs were found in ‘Glutathione transferase activity’ (GO:0004364), ‘Chitinase activity’ (GO:0004568), ‘Nutrient reservoir activity’ (GO:0045735), and ‘Manganese ion binding’ (GO:0030145), respectively (Figure 3).

**Figure 4:**
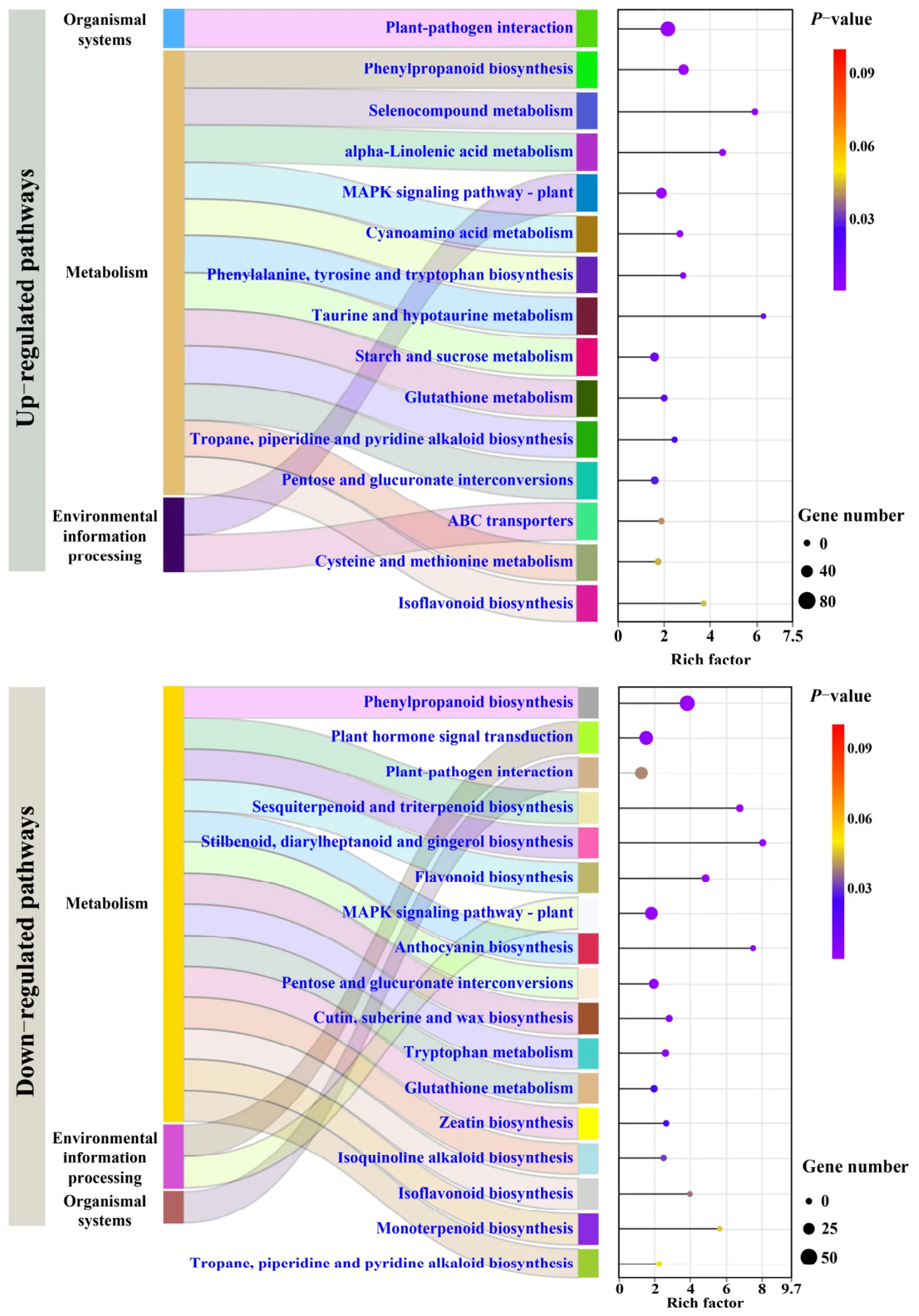
Sankey diagram of upregulated and downregulated KEGG pathways. The top 15 upregulated and downregulated pathways were displayed.

To identify the pathways in which DEGs participate, we then performed KEGG pathway analysis. In total, we identified 15 upregulated pathways and 17 downregulated pathways (Figure 4). The ‘plant-pathogen interaction’ pathway, categorized under organismal systems, contained the largest number of DEGs with both upregulated and downregulated expression profiles. In addition, the ‘phenylpropanoid biosynthesis’, ‘MAPK signaling pathway-plant’, ‘tropane, piperidine and pyridine alkaloid biosynthesis’, ‘glutathione metabolism’, ‘pentose and glucuronate interconversions’, and ‘isoflavonoid biosynthesis’ pathways harbored both upregulated and downregulated DEGs (Figure 4). Several upregulated DEGs were exclusively enriched in the ‘selenocompound metabolism’, ‘α-linolenic acid metabolism’, ‘cyanoamino acid metabolism’, ‘phenylalanine, tyrosine and tryptophan biosynthesis’, and ‘starch and sucrose metabolism’ pathways. Furthermore, numerous DEGs were enriched in downregulated pathways, including ‘plant hormone signal transduction’, ‘flavonoid biosynthesis’, ‘anthocyanin biosynthesis’, and ‘cutin, suberine and wax biosynthesis’ (Figure 4). These results suggest that these pathways may participate in the host response to fungal infection.

### 3.4. Analysis of DEGs Isolated from GO and KEGG Enrichments

**Figure 5:**
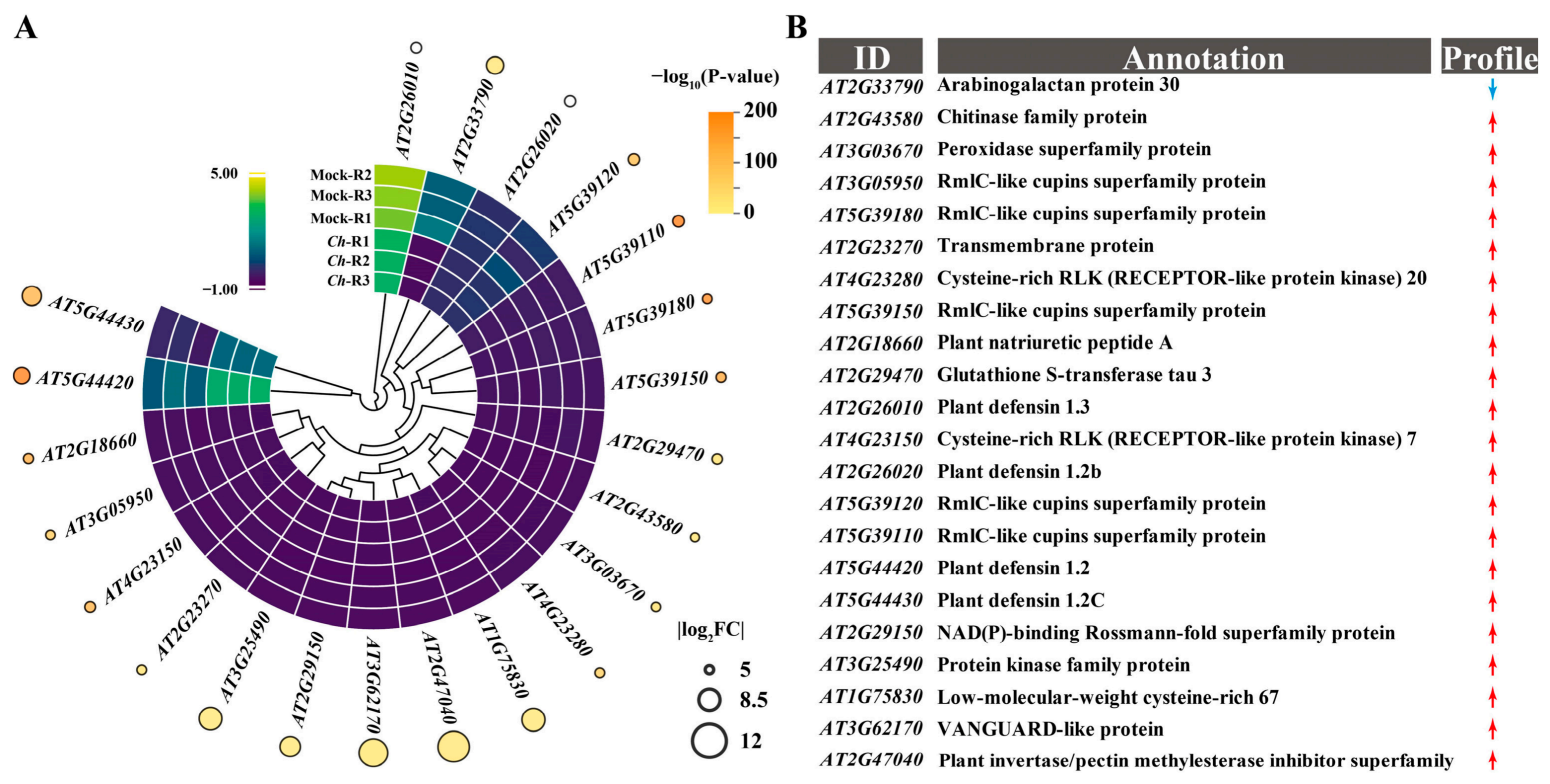
The analysis of expression profiles of specific DEGs. (**A**) Expression heatmap of DEGs isolated from GO and KEGG enrichment analysis. The expression fold change of each gene was displayed using circles. The expression levels of each gene were normalized based on the FPKM value; (**B**) the annotations of specific DEGs. The expression profile of each DEG was displayed using arrows. Red arrows denote upregualtion and blue arrows denote downregulation.

After GO and KEGG analysis, we compiled all DEGs. After removing redundant DEGs identified in both GO and KEGG enrichments, a total of 94 DEGs were retained. To further narrow the candidate list, we filtered DEGs with |log_2_FC| greater than 5. Finally, 22 DEGs were obtained (Figure 5A). A comparison of normalized transcript levels across Mock and *Ch*-treated replicates revealed that nearly all tested DEGs exhibited pronounced transcriptional induction upon *Ch* infection, as evidenced by elevated expression intensities in *Ch* samples relative to the Mock group (Figure 5). Among these genes, only AT2G33790 (arabinogalactan protein 30, AGP30) showed consistent transcriptional repression, whereas all other genes were induced by *Ch* infection. Four RmlC-like cupins genes (AT3G05950, AT5G39180, AT5G39150, and AT5G39110), four plant defensin paralogs (AT2G26010, AT2G26020, AT5G44420, and AT5G44430), and two CRK receptors (AT4G23280 and AT4G23150) exhibited comparable expression intensities across all samples and similar |log_2_FC| magnitudes, indicating coordinated co-upregulation during the pathogen response. Three loci, including AT3G62170 (VANGUARD-like protein), AT2G47040 (pectin modification inhibitor protein), and AT1G75830 (low-molecular-weight cysteine-rich 67, LCR67), had the highest fold changes. In addition, genes encoding the peroxidase superfamily protein (AT3G03670), glutathione transferase tau 3 (AT2G29470), chitinase family protein (AT2G43580), and protein kinase family protein (AT3G25490) also showed clearly altered expression profiles (Figure 5).

### 3.5. Expression Validation of DEGs

**Figure 6:**
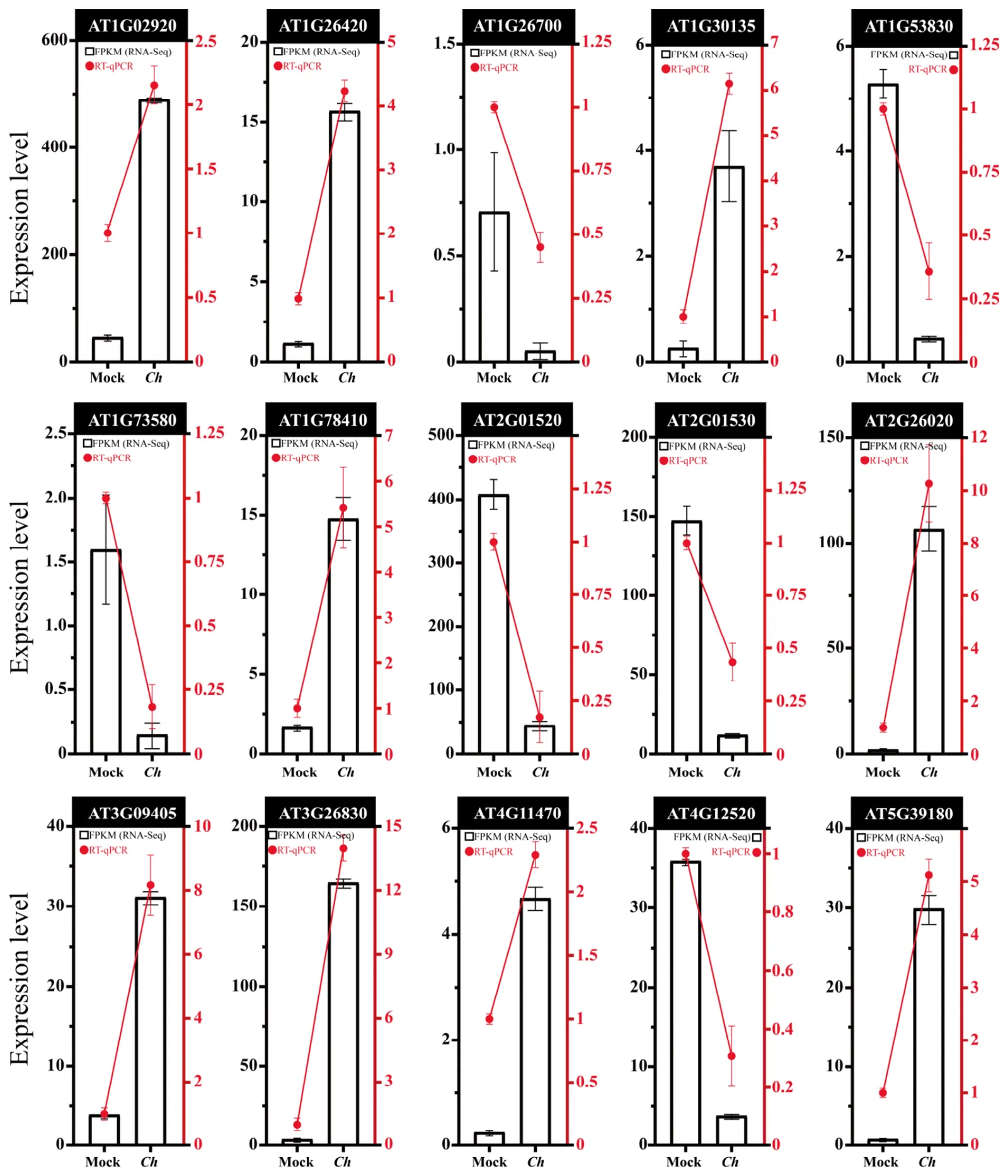
Validation of DEGs. For qRT-PCR results, bars indicate Mean ± SD; for FPKM values, bars indicate errors originating from three biological replicates.

To further verify the reliability of the transcriptome data, we performed quantitative real-time PCR (qRT-PCR) to analyze the expression levels of candidate genes across different samples. We selected 15 genes from these candidates. The results showed that the expression trends from transcriptome (FPKM) and qRT-PCR data were consistent for each gene (Figure 6), confirming the high reliability of the transcriptome data.

### 3.6. Comparative Analysis of Metabolome

**Figure 7:**
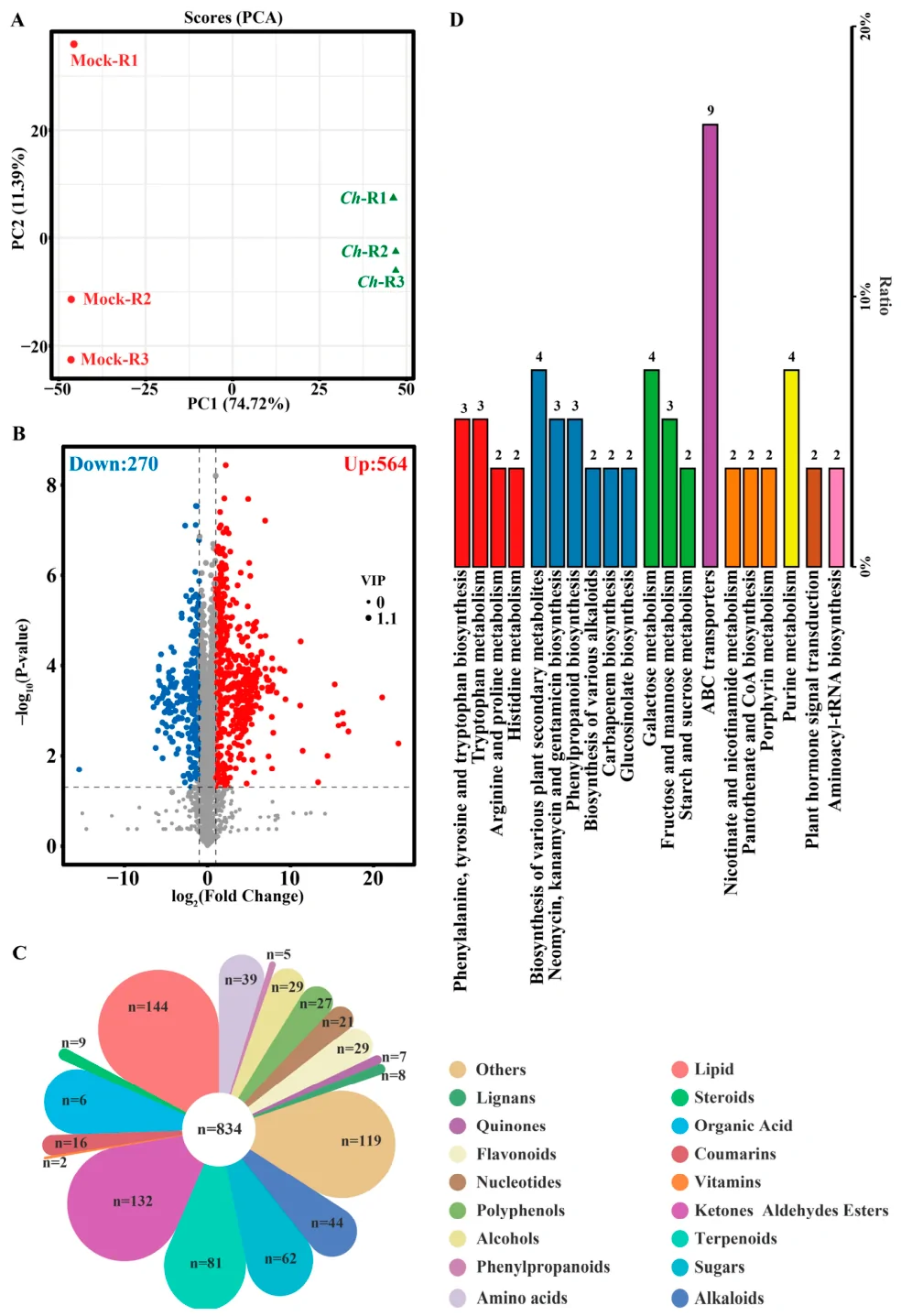
Metabolomic profiling and differentially accumulated metabolite (DAM) analysis between Mock and *Ch*-infected samples. (**A**) Principal component analysis (PCA) score plot of all samples; (**B**) volcano plot of DAMs; (**C**) the statistics of different types of DAMs; (**D**) KEGG pathway enrichment analysis of DAMs.

Plant metabolites, particularly secondary metabolites, are the cornerstone of plant immunity. To investigate metabolite changes in Mock and *Ch*-infected samples, we performed a comparative metabolomic analysis. As shown in Figure 7A, PCA revealed that PC1 and PC2 accounted for 74.72% and 11.39% of the total metabolic variation, respectively, with a cumulative contribution of 86.11%. Samples of the Mock control group and *Ch* treatment group were clearly separated along the PC1 axis, and biological replicates within each group clustered tightly, indicating reliable experimental reproducibility and that *Ch* treatment profoundly reshaped the global metabolome. Volcano plot analysis identified a total of 834 significantly differentially accumulated metabolites (DAMs) between Mock and *Ch* groups, including 564 significantly upregulated and 270 significantly downregulated DAMs (Figure 7B). Classification of DAMs demonstrated that the predominant compounds included coumarins, ketones, aldehydes, esters, terpenoids, sugars, alkaloids, amino acids, flavonoids, and polyphenols. Notably, phenylpropanoid-derived secondary metabolites such as coumarins, flavonoids, and polyphenols represented an important group among DAMs, while terpenoids, synthesized via the MEP and mevalonate pathways, constituted another abundant metabolite class (Figure 7C). Among the 29 identified flavonoids, 12 were upregulated, and 17 were downregulated. KEGG enrichment analysis indicated that DAMs were significantly enriched in pathways including ABC transporters, biosynthesis of various plant secondary metabolites, phenylpropanoid biosynthesis, phenylalanine, tyrosine and tryptophan biosynthesis, starch and sucrose metabolism, galactose metabolism, amino acid metabolism, and plant hormone signal transduction (Figure 7D). These results demonstrated that *Ch* treatment triggers extensive metabolic reprogramming.

### 3.7. Integrated Analysis of Transcriptome and Metabolome

**Figure 8:**
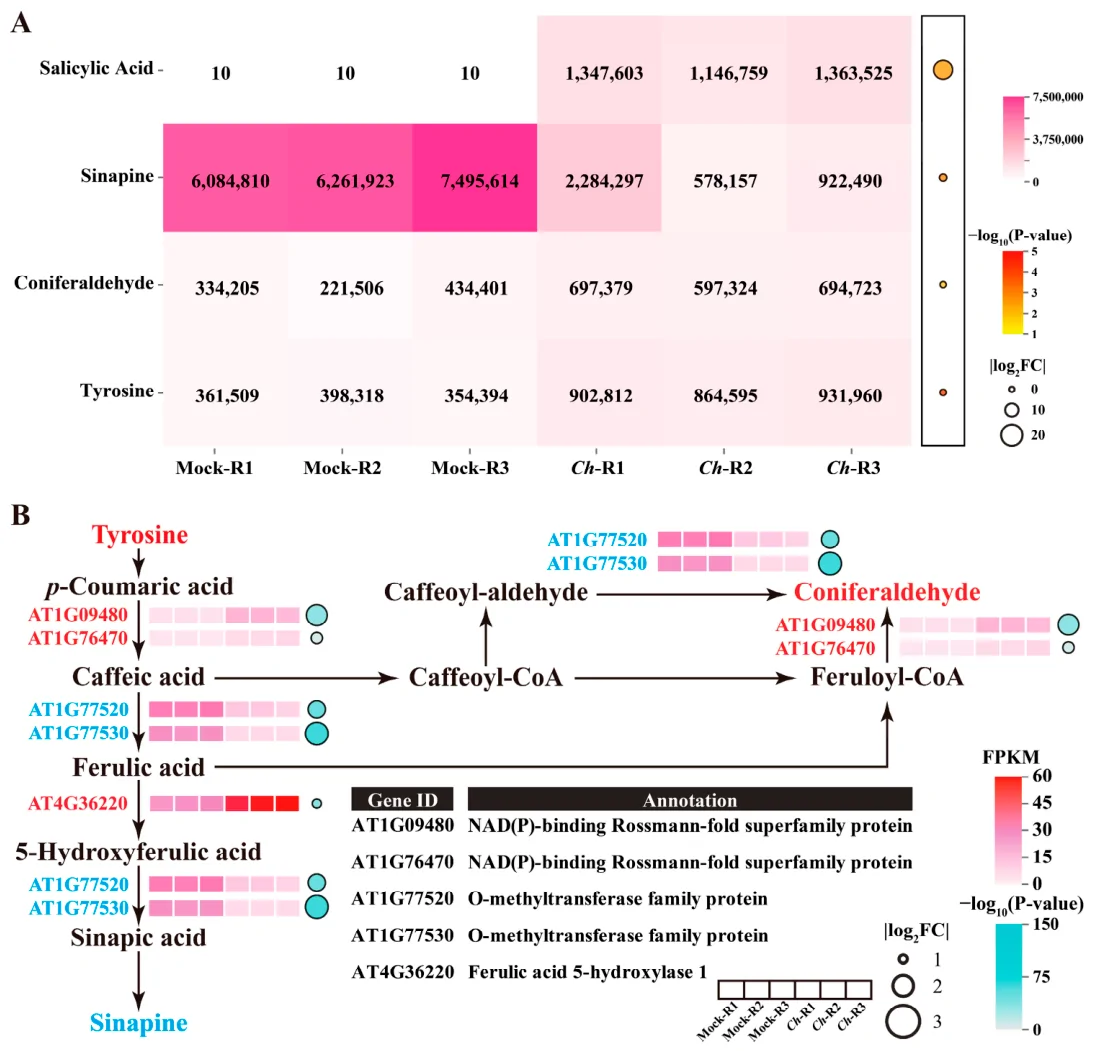
Integrated transcriptomic and metabolomic analysis of the phenylpropanoid biosynthesis pathway. (**A**) The accumulation heatmaps of DAMs directly or indirectly participating in the phenylpropanoid biosynthesis pathway. Note: The salicylic acid signal was below the instrumental detection limit (signal intensity = 10) in Mock samples; a constant value of 10 was imputed for fold change calculation. (**B**) The diagram showed the patterns of the DEGs and DAMs in the phenylpropanoid biosynthesis pathway. For the gene ID and metabolites, red means upregulation and blue means downregulation. The annotations of DEGs are shown as well. The boxes denote expression levels of DEGs and circles refer to expression fold changes. The expression intensity is shown using FPKM values.

Based on the results of transcript-dependent KEGG analysis and metabolome-based KEGG analysis, we found that several DEGs and DAMs were enriched in the phenylpropanoid biosynthesis pathway (Figure 4 and Figure 5), which is a pathway that massively synthesizes phenolic defensive secondary metabolites. Furthermore, salicylic acid (SA) synthesis can utilize phenylalanine, which is an upstream precursor of the phenylpropanoid pathway, and cooperatively regulates plant defense together with the phenylpropanoid metabolic network. Given these findings, we then found that three DAMs, including tyrosine, coniferaldehyde, and sinapine, were upregulated and downregulated after *Ch* infection, respectively. In addition, the content of SA was significantly increased (log_2_FC = 16.97) due to *Ch* infection (Figure 8A).

Next, we jointly analyzed the transcriptome and metabolome data, which covered the responsive profiles of key genes and metabolites involved in the sinapine biosynthesis branch of the phenylpropanoid pathway. Tyrosine, serving as the upstream precursor of this pathway, exhibited significantly elevated abundance under *Ch* treatment to continuously supply carbon skeletons for phenylpropanoid metabolism. Key structural genes, including AT4G36220 (ferulic 5-hydroxylase 1, F5H1) and O-methyltransferase-encoding genes AT1G77520 and AT1G77530, were markedly upregulated upon *Ch* exposure, facilitating the conversion of ferulic acid into 5-hydroxyferulic acid and sinapine. Meanwhile, increased expression of two NAD(P)-binding Rossmann-fold superfamily protein-encoding genes AT1G09480 and AT1G76470 promoted the flux of intermediate metabolites toward coniferaldehyde. Integrated with metabolite quantification data, we observed that although the genes responsible for sinapine biosynthesis were transcriptionally activated, the final product sinapine was significantly reduced after *Ch* treatment, accompanied by massive accumulation of coniferaldehyde (Figure 8B).

### 3.8. Analysis of Transcription Factors

**Figure 9:**
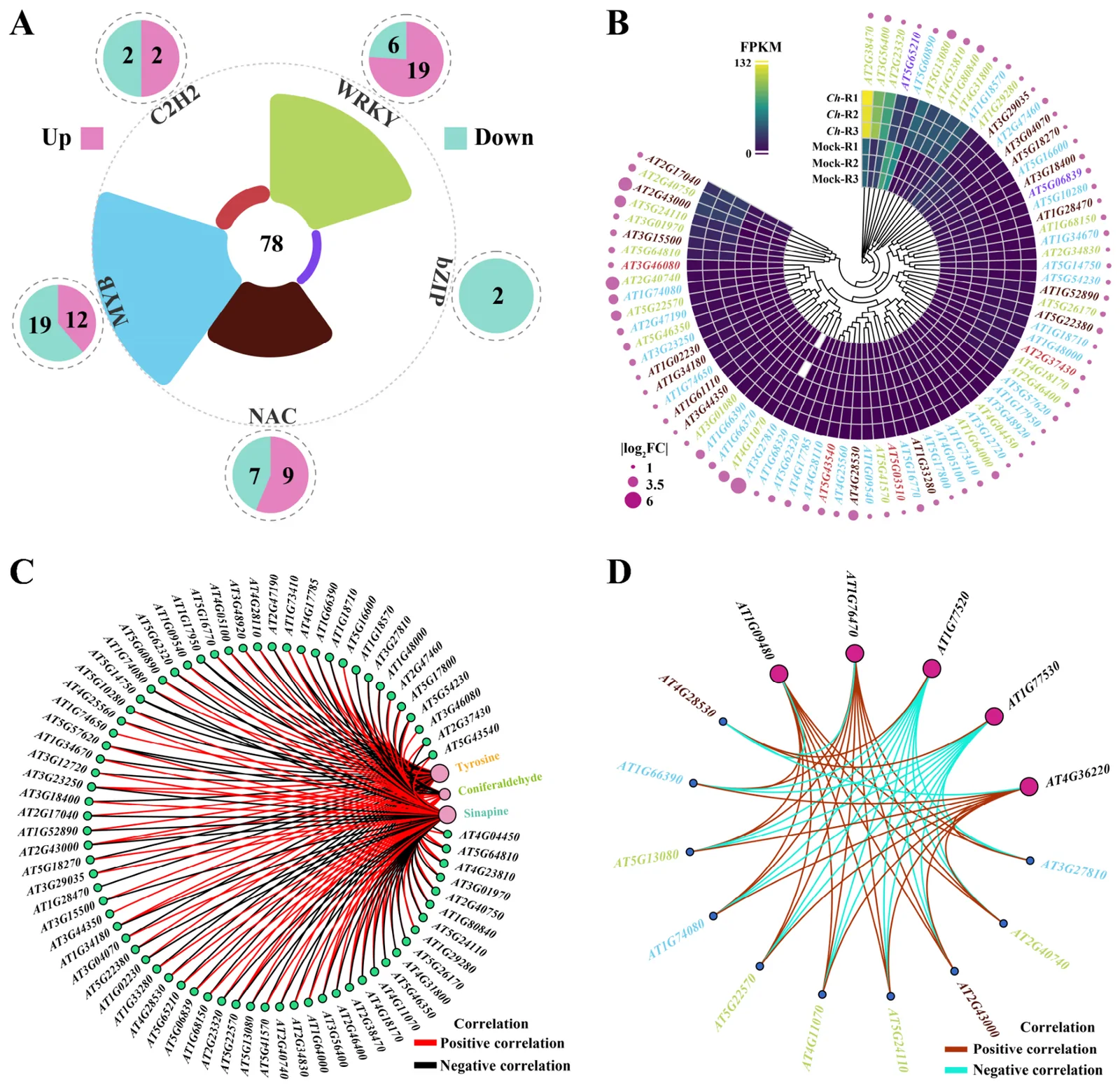
Analysis of the type of TFs and the correlation between TFs and DEGs and DAMs in the phenylpropanoid biosynthesis pathway. (**A**) The type and number of DE-TFs. The number of upregulated and downregulated TFs are also shown. (**B**) The expression heatmap of all DE-TFs based on their FPKM values. Each type of TF was displayed using different colors in line with the colors in (**A**). The expression fold change of each DE-TF is displayed using circles. (**C**) Correlation between DE-TFs and three DAMs in the phenylpropanoid biosynthesis pathway. (**D**) Correlation between 10 DE-TFs with |log_2_FC| over three and five enzyme-encoding genes in the phenylpropanoid biosynthesis pathway.

Transcription factors (TFs) function as central regulatory hubs linking upstream immune signals to downstream transcriptional reprogramming and metabolic defense networks in plant immunity. To identify which TFs respond to *Ch* infection, we characterized differentially expressed TFs (DE-TFs). A total of 78 DE-TFs were identified under *Ch* treatment, mainly belonging to the WRKY, MYB, and NAC families (Figure 9A). The WRKY TF set included more upregulated genes, whereas most differentially expressed MYB genes were downregulated. The circular heatmap of DE-TF expression profiles confirmed that most DE-TFs were transcriptionally activated upon *Ch* exposure (Figure 9B). TF–metabolite correlation networks revealed that numerous TFs exhibited significant positive correlations with tyrosine and coniferaldehyde, while showing negative correlations with sinapine, which accounts for the redistribution of metabolic flux within the phenylpropanoid pathway at the transcriptional level (Figure 9C; Table S3). We then selected 10 DE-TFs with |log_2_FC| greater than three and conducted correlation analysis with five enzyme-encoding genes in the phenylpropanoid biosynthesis pathway. Subnetwork analysis identified hub TFs that were significantly correlated with key structural genes involved in sinapine and coniferaldehyde biosynthesis, such as AT4G36220, AT1G77520, and AT1G77530 (Figure 9D; Table S4). These results demonstrate that TFs may remodel carbon allocation in secondary metabolism by modulating the transcription of phenylpropanoid biosynthetic genes, promoting accumulation of the lignin precursor coniferaldehyde and thereby contributing to plant immune defense.

## 4. Discussion

### 4.1. Ch Infection Reprogrammed Transcriptome and Metabolome Profiles

*Colletotrichum* spp. rank among the top ten phytopathogenic fungi worldwide that pose severe threats to agricultural crops [35]. As a hemibiotrophic fungus, *Ch* can infect Brassicaceae plants, including the model plant *Arabidopsis*. This infection process involves sophisticated molecular interactions and precise regulatory mechanisms. In this study, transcriptomic analysis was performed to systematically uncover the extensive reprogramming of host gene expression triggered by *Ch* infection and further elucidate the intrinsic correlation between transcriptional reprogramming and the suppression of growth.

Based on DEG analysis and enrichment analysis, we found that host plants rapidly activate a complex network of defense responses, which is manifested at the transcriptomic level by the activation of numerous defense-related genes. In addition, the burst of reactive oxygen species (ROS) is an important marker of the early plant defense response. Our study also detected significantly upregulated expression of genes related to ROS production and scavenging, such as peroxidase superfamily genes and glutathione S-transferase tau 3. ROS not only exerts direct toxicity to pathogens but also acts as signaling molecules to activate downstream defense responses [35,36]. Thus, the GO terms ‘Peroxidase activity’ and ‘Oxidoreductase activity’ were significantly enriched in this study.

Plants dynamically regulate the synthesis and accumulation of metabolites, coordinate immune signaling pathways, balance growth and development with disease resistance and defense, and exert core regulatory functions in host–pathogen interactions [37]. Over 800 metabolites, including several defense-related metabolites, showed altered accumulation levels after *Ch* infection, suggesting that the host plants respond positively to pathogen infection via reprogramming the metabolic profile. For instance, we found SA content in *Ch*-treated samples was markedly higher, indicating that the signaling pathway of SA was activated during *Ch* infection, which was in line with a previous study [38].

Overall, transcriptomic and metabolic reprogramming induced by infection with *Ch* constitutes the direct molecular basis for plant growth inhibition in this study. Plants must trade off between growth and defense under limited resources. When threatened by pathogens, plants redistribute substantial amounts of energy and metabolites from growth and developmental processes to defense responses. This redistribution of resources is manifested at the transcriptomic and metabolic level as the general downregulation of growth-related genes and the significant upregulation of defense-related genes and metabolites. Our results fully support this opinion.

### 4.2. Phenylpropanoid Metabolism Might Have Key Function During Ch Infection in Host Plants

During long-term evolution, plants have evolved complex and sophisticated defense systems to cope with diverse biotic and abiotic stresses [39]. Among these processes, secondary metabolism plays a vital role in plant immunity [40]. Our results revealed that *Ch* infection significantly activates the phenylpropanoid pathway, with multiple DEGs and DAMs enriched within this pathway. This finding not only reveals the mode of action of the elicitor but also further confirms that phenylpropanoid metabolism acts as a core hub of plant innate immunity [41].

Tyrosine acts as a biosynthetic precursor for numerous defensive secondary metabolites and provides the material basis for plants to establish chemical defense barriers [42,43]. The canonical phenylpropanoid pathway in *Arabidopsis* uses phenylalanine as the primary upstream precursor. A tyrosine-derived shortcut for monolignol biosynthesis exists exclusively in grasses that encode bifunctional phenylalanine/tyrosine ammonia-lyase (PTAL) [44], and this route is absent in *Arabidopsis*. Tyrosine also serves as a precursor for isoquinoline alkaloid biosynthesis in maize, a pathway independent of phenylpropanoid metabolism [45]. In *Arabidopsis*, pathogen-induced SA is predominantly synthesized via the ICS1-dependent isochorismate pathway, whereas the phenylpropanoid pathway constitutes a secondary source of SA. In addition, studies in rice have shown that during the response of resistant varieties to *Magnaporthe oryzae* infection, differentially expressed long non-coding RNAs (lncRNAs) are significantly enriched in the biosynthetic pathways of phenylalanine, tyrosine, and tryptophan. These amino acids serve as SA precursors, highlighting the critical role of the tyrosine metabolic pathway in the disease resistance regulatory network [46]. Accordingly, the elevated SA abundance in *Ch*-treated samples was mainly attributable to the activation of phenylpropanoid metabolism.

Coniferaldehyde is a key intermediate in the biosynthetic pathway of lignin monomers and acts as the direct precursor for guaiacyl lignin (G-lignin) synthesis. Lignin is a major structural component of plant secondary cell walls. By increasing the mechanical strength and hydrophobicity of cell walls, lignin forms a robust physical barrier that effectively restricts the invasion and spread of phytopathogens [47]. The accumulation of coniferaldehyde, the key precursor of guaiacyl lignin, together with reduced sinapate ester abundance and the differential expression of AT1G76470 (encoding a cinnamoyl-CoA reductase family protein), suggests that *Ch* infection may redirect phenylpropanoid metabolic flux toward lignin biosynthesis. This metabolic reprogramming coincides with the downregulation of cutin, suberin, and wax biosynthetic pathways. However, since direct lignin quantification or histochemical staining was not performed in the present study, this hypothesis of metabolic flux redirection requires further experimental validation in future work, as demonstrated in prior research integrating transcriptional profiling with lignin histochemistry [48].

Sinapine is the choline ester of sinapic acid and is widely distributed across Brassicaceae species. Sinapic acid is produced through the sinapate branch of the phenylpropanoid pathway, derived from sinapaldehyde and sinapyl alcohol; sinapyl alcohol functions as the monomer for syringyl lignin (S-lignin) biosynthesis [47]. Traditionally, sinapine has been regarded as a storage form of sinapic acid that is mobilized during seed germination. Nevertheless, accumulating evidence indicates that such soluble phenolic compounds exert active functions in plant–environment interactions. In this study, we observed a marked reduction in sinapine abundance following *Ch* infection. As a phenolic metabolite with strong antioxidant capacity, sinapine may scavenge excess reactive oxygen species (ROS) to maintain intracellular redox homeostasis and alleviate oxidative cellular damage caused by the pathogen-triggered oxidative burst. Our multi-omics data uncovered regulatory discordance in sinapine metabolism: genes associated with sinapine biosynthesis were transcriptionally upregulated upon infection, while the abundance of the end-product sinapine decreased, accompanied by coniferaldehyde accumulation. This pattern indicates that newly synthesized sinapine is rapidly consumed, most likely for ROS scavenging during host defense, rather than that sinapine biosynthesis is suppressed by the pathogen.

In summary, our results demonstrate that activation of the phenylpropanoid metabolic pathway via endogenous or exogenous elicitors represents an effective strategy to enhance plant disease resistance. This process involves multilayered, network-wide regulation of plant defense systems and is tightly linked to hormone signaling pathways.

### 4.3. TFs Might Play an Important Role During Plant Responses to Ch Infection

As the core of plant secondary metabolism, the phenylpropanoid metabolic pathway plays a vital role in defense responses [20,49]. Multiple compounds synthesized via this pathway, such as lignin, flavonoids, and phenolic acids, can not only reinforce cell walls as physical barriers but also participate in immune responses as signaling molecules or direct antimicrobial substances [49,50]. Nevertheless, how plants precisely regulate this complex metabolic network to respond to specific pathogen threats while avoiding unnecessary growth costs remains a central question in plant immunology research. TFs, acting as “switches” for gene expression, fulfill central roles in this process [51]. In this study, we identified 79 DE-TFs, including members of the WRKY, NAC, MYB, C2H2, and bZIP families.

Numerous studies have demonstrated that multiple TF families, especially MYB and WRKY, act as master regulators of plant secondary metabolism and stress responses [52,53]. As one of the largest transcription factor families in plants, MYBs are extensively involved in modulating the phenylpropanoid metabolic pathway [54,55]. They can function as activators or repressors to fine-tune metabolic flux toward distinct pathway branches. For instance, during strawberry fruit ripening, FaMYB10 modulates the flavonoid/phenylpropanoid pathway to promote anthocyanin accumulation [56]. In pepper, CcMYB4-12 acts as a repressor and regulates lignin and capsaicin biosynthesis by inhibiting transcription of *CcPAL2* [57]. AtMYB122 contributes to restricting *Ch* biotrophic growth, possibly through specific regulation of glucosinolate (GSL) pathways in epidermal cells [58]. Meanwhile, the WRKY TF family is recognized as a core component of the plant immune signaling network [59,60]. To date, accumulating evidence indicates that WRKY TFs also directly participate in the regulation of secondary metabolic pathways. For example, cotton GhWRKY41 (the homolog of AtWRKY41) can directly activate *GhC4H* and *Gh4CL*, which encode key enzymes in the phenylpropanoid metabolic pathway, thereby enhancing resistance to Verticillium wilt [61]. In this study, we found that MYB and WRKY TFs exhibited strong correlations with the enzymes F5H1 and O-methyltransferase, implying that MYB and WRKY TFs may directly regulate the biosynthesis of tyrosine, coniferaldehyde, and sinapine. These findings further reveal the direct involvement of MYB and WRKY TFs in integrating immune signals and metabolic reprogramming.

NAC TFs exert dual functions in plant immune responses. They can serve as positive regulators to activate defense genes or as negative regulators to suppress excessive immune activation, thereby maintaining the balance between immunity and growth [62,63]. In recent years, accumulating evidence from combined transcriptomic and metabolomic analyses has shown that altered expression of NAC TFs is tightly linked to global regulation of the phenylpropanoid metabolic pathway. For example, in *Trichoderma*-induced resistance to Fusarium wilt in melon, upregulation of NAC domain proteins coincides with significant enrichment of the phenylpropanoid biosynthesis pathway [64]. Similarly, synergistic response patterns between NAC TFs and the phenylpropanoid metabolic pathway have been documented in multiple plant species including poplar, quinoa, and maize under abiotic stresses such as salinity and waterlogging [65,66,67]. In our study, 16 NAC TFs displayed strong correlations with metabolites of the phenylpropanoid pathway, and two NAC TFs showed positive (AT2G43000) or negative (AT4G28530) associations with enzyme-encoding genes. A prior study reported that the NAC TF NST1/SND1 in the model legume *Medicago truncatula* can induce *F5H* expression [47]. Collectively, these lines of evidence suggest that the NAC TFs identified here may potentially regulate *F5H* expression. Accordingly, we propose that NAC TFs likely act as key upstream switches governing the phenylpropanoid metabolic pathway during *Ch* infection.

bZIP TFs are well known for their broad roles in plant hormone signaling and stress responses [68]. Our study reveals that specific bZIP members respond to *Ch* infection, and their expression patterns are positively or negatively correlated with the enzyme genes and metabolites we identified within the phenylpropanoid pathway. This is consistent with previous reports across diverse plants showing that bZIP family members are markedly induced upon pathogen attack, salt stress, or drought stress [41,69,70]. For example, in grapevine, VvHY5 has been characterized as a transcriptional repressor of key enzyme genes in ethylene biosynthesis, and ethylene signaling is closely associated with phenylpropanoid metabolism [71]. Therefore, bZIP transcription factors may serve as hubs that integrate multiple stress and hormone signals, relay these signals to the phenylpropanoid metabolic pathway, and thereby trigger corresponding defense responses.

C2H2 zinc finger proteins represent one of the largest TF families in plants and participate in nearly all biological processes, ranging from growth and development to stress responses [72]. Despite their functional diversity, relatively few studies have addressed their specific roles in secondary metabolism regulation. Two C2H2 TFs identified in this study showed pronounced expression changes upon activation of plant immunity and exhibited strong positive or negative relationships with the accumulation of sinapine, coniferaldehyde, and tyrosine, suggesting that C2H2 TFs may participate in defense against *Ch*.

## 5. Conclusions

In this study, we performed integrated transcriptome and metabolome analysis to characterize changes in host plant genes and metabolites upon *Ch* infection. Our main finding confirms the essential role of phenylpropanoid metabolism during *Ch* infection. The altered accumulation of tyrosine, coniferaldehyde, and sinapine may be an important factor underlying the ability of *Ch* to infect host plants and suppress host growth. In addition, five enzyme-encoding genes were identified, and they may serve as novel targets for improving resistance against *Ch* infection. Furthermore, 78 TFs belonging to the WRKY, MYB, NAC, C2H2, and bZIP families were also screened. Several of these TFs exhibited high expression fold changes following *Ch* infection and strong positive or negative correlations with enzyme-encoding genes in the phenylpropanoid metabolic pathway, indicating that these TFs may participate in the regulation of phenylpropanoid metabolism. Although further experiments are required to fully elucidate the regulatory network between TFs and enzyme-encoding genes, our results provide valuable genetic resources and molecular targets for future investigations into plant–pathogen interactions. Meanwhile, this study has several limitations. First, multi-omics profiling was conducted using only a single sampling time point, which fails to capture the dynamic progression of plant responses to *Ch* colonization. Second, although we hypothesize a potential growth–defense trade-off during this host–pathogen interaction, this idea remains unvalidated in the present study and requires dedicated follow-up experiments. Third, although individual seedlings were used as the experimental unit for data collection, multiple seedlings on the same plate shared identical medium and inoculum sources. Potential plate-level confounding effects could not be fully excluded, which should be considered when interpreting the present results.

## Data Availability

The data presented in this study are available upon request.

## Supplementary Materials

The following supporting information can be downloaded at https://www.mdpi.com/article/10.3390/life16091505/s1, Figure S1: Phylogenetic tree of *Colletotrichum* species constructed based on nucleotide sequences. Bootstrap values are shown at the nodes. *Ch* represents the strain isolated in this study, and other reference sequences were retrieved from the NCBI GenBank database; Table S1: The primers used in this study; Table S2: Sequencing data statistics and the result of sequence alignment between the sample sequencing data and the selected reference genome; Table S3: The correlation coefficients and *p*-values between DF-TFs and 3 DAMs; Table S4: The correlation coefficients and *p*-values between DE-TFs and five enzyme-encoding genes in the phenylpropanoid biosynthesis pathway.

## Abbreviations

The following abbreviations are used in this manuscript:

AGP30	Arabinogalactan Protein 30
BP	Biological Process
CAD	Collision-Activated Dissociation
CC	Cellular Component
CE	Collision Energy
*Ch*	*Colletotrichum higginsianum*
CRK	Cysteine-Rich Receptor-Like Kinase
CUR	Curtain Gas
DAM	Differentially Accumulated Metabolite
DE-TF	Differentially Expressed Transcription Factor
DP	Declustering Potential
dpi	Days Post-Inoculation
F5H1	Ferulate 5-Hydroxylase 1
FC	Fold Change
FPKM	Fragments Per Kilobase of Transcript per Million Mapped Fragments
GO	Gene Ontology
HMDB	Human Metabolome Database
HY5	Elongated Hypocotyl 5
KEGG	Kyoto Encyclopedia of Genes and Genomes
LCR67	Low-Molecular-Weight Cysteine-Rich 67
LIT	Linear Ion Trap
lncRNA	Long Non-Coding RNA
MAPK	Mitogen-Activated Protein Kinase
MF	Molecular Function
MRM	Multiple Reaction Monitoring
MS	Murashige and Skoog medium
OPLS-DA	Orthogonal Partial Least Squares-Discriminant Analysis
PCA	Principal Component Analysis
PDA	Potato Dextrose Agar
QC	Quality Control
QQQ	Triple Quadrupole
qRT-PCR	Quantitative Reverse Transcription Polymerase Chain Reaction
ROS	Reactive Oxygen Species
SA	Salicylic Acid
TF	Transcriptional Factor
TYDC1	Tyrosine Decarboxylase 1
UPLC-MS/MS	Ultra-Performance Liquid Chromatography-Tandem Mass Spectrometry
VIP	Variable Importance in Projection
WT	Wild Type

## References

[1] Khodadadi F., Giroux E., Bilodeau G.J., Jurick W.M., Aćimović S.G. (2023). Genomic resources of four *Colletotrichum* species (*C. fioriniae*, *C. chrysophilum*, *C. noveboracense*, and *C. nupharicola*) threatening commercial apple production in the eastern United States. Mol. Plant-Microbe Interact..

[2] Jayawardena R., Hyde K., Damm U., Cai L., Liu M., Li X., Zhang W., Zhao W., Yan J. (2016). Notes on currently accepted species of *Colletotrichum*. Mycosphere.

[3] Cannon P.F., Damm U., Johnston P.R., Weir B.S. (2012). *Colletotrichum*–current status and future directions. Stud. Mycol..

[4] Jayawardena R.S., Bhunjun C.S., Hyde K.D., Gentekaki E., Itthayakorn P. (2021). *Colletotrichum*: Lifestyles, biology, morpho-species, species complexes and accepted species. Mycosphere.

[5] Chen Y.P., Wu T., Tian W.H., Ilyukhin E., Hyde K.D., Maharachchikumbura S.S.N. (2022). Comparative genomics provides new insights into the evolution of *Colletotrichum*. Mycosphere.

[6] Liu F., Ma Z.Y., Hou L.W., Diao Y.Z., Wu W.P., Damm U., Song S., Cai L. (2022). Updating species diversity of *Colletotrichum*, with a phylogenomic overview. Stud. Mycol..

[7] Perfect S.E., Hughes H.B., O’Connell R.J., Green J.R. (1999). *Colletotrichum*: A model genus for studies on pathology and fungal–plant interactions. Fungal Genet. Biol..

[8] Dean R., Van Kan J.A.L., Pretorius Z.A., Hammond-Kosack K.E., Di Pietro A., Spanu P.D., Rudd J.J., Dickman M., Kahmann R., Elis J. (2012). The top 10 fungal pathogens in molecular plant pathology. Mol. Plant Pathol..

[9] Liu X., Li B., Cai J., Zheng X., Feng Y., Huang G. (2018). *Colletotrichum* species causing anthracnose of rubber trees in China. Sci. Rep..

[10] Ling J.F., Song X.B., Xi P.G., Cheng B.P., Cui Y.P., Chen X., Peng A.T., Jiang Z.D., Zhang L.H. (2019). Identification of *Colletotrichum siamense* causing litchi pepper spot disease in mainland China. Plant Pathol..

[11] Qin L.P., Zhang Y., Su Q., Chen Y., Nong Q., Xie L., Yu G.M., Huang S.L. (2019). First report of anthracnose of *Mangifera indica* caused by *Colletotrichum scovillei* in China. Plant Dis..

[12] Khodadadi F., González J.B., Martin P.L., Giroux E., Bilodeau G.J., Peter K.A., Doyle V.P., Aćimović S.G. (2020). Identification and characterization of *Colletotrichum* species causing apple bitter rot in New York and description of *C. noveboracense* sp. nov. Sci. Rep..

[13] Yan Y., Yuan Q., Tang J., Huang J., Hsiang T., Wei Y., Zheng L. (2018). *Colletotrichum higginsianum* as a Model for Understanding Host–Pathogen Interactions: A Review. Int. J. Mol. Sci..

[14] Huser A., Takahara H., Schmalenbach W., O’Connell R. (2009). Discovery of pathogenicity genes in the crucifer anthracnose fungus *Colletotrichum higginsianum*, using random insertional mutagenesis. Mol. Plant-Microbe Interact..

[15] Takahara H., Hacquard S., Kombrink A., Hughes H.B., Halder V., Robin G.P., Hiruma K., Neumann U., Shinya T., Kombrink E. (2016). *Colletotrichum higginsianum* extracellular LysM proteins play dual roles in appressorial function and suppression of chitin-triggered plant immunity. New Phytol..

[16] Robin G.P., Kleemann J., Neumann U., Cabre L., Dallery J.F., Lapalu N., O’Connell R.J. (2018). Subcellular Localization Screening of Colletotrichum higginsianum Effector Candidates Identifies Fungal Proteins Targeted to Plant Peroxisomes, Golgi Bodies, and Microtubules. Front. Plant Sci..

[17] O’Connell R.J., Thon M.R., Hacquard S., Amyotte S.G., Kleemann J., Torres M.F., Damm U., Buiate E.A., Epstein L., Alkan N. (2012). Lifestyle transitions in plant pathogenic *Colletotrichum* fungi deciphered by genome and transcriptome analyses. Nat. Genet..

[18] Seufer J., Speer D., Aus den Erlen R., Glawischnig E., Voll L.M. (2026). Response of the *Arabidopsis* indolic secondary metabolite network to infection with *Colletotrichum higginsianum*. Front. Fungal Biol..

[19] Ritter H., Schulz G.E. (2004). Structural basis for the entrance into the phenylpropanoid metabolism catalyzed by phenylalanine ammonia-lyase. Plant Cell.

[20] Dong N.Q., Lin H.X. (2021). Contribution of phenylpropanoid metabolism to plant development and plant-environment interactions. J. Integr. Plant Biol..

[21] Ninkuu V., Aluko O.O., Yan J., Zeng H., Liu G., Zhao J., Li H., Chen S., Dakora F.D. (2025). Phenylpropanoids metabolism: Recent insight into stress tolerance and plant development cues. Front. Plant Sci..

[22] Douglas C. (1996). Phenylpropanoid metabolism and lignin biosynthesis: From weeds to trees. Trends Plant Sci..

[23] Dixon R.A., Paiva N.L. (1995). Stress-Induced Phenylpropanoid Metabolism. Plant Cell.

[24] Castelluccio C., Paganga G., Melikian N., Bolwell G.P., Pridham J., Sampson J., Rice-Evans C. (1995). Antioxidant potential of intermediates in phenylpropanoid metabolism in higher plants. FEBS Lett..

[25] Dong Q., Huang T., Zhou C., Wan X., He X., Miao P., Cheng H., Wang X., Yu H., Hu M. (2026). Nano-priming with selenium nanoparticles reprograms seed germination, antioxidant defense, and phenylpropanoid metabolism to enhance *Fusarium graminearum* resistance in maize seedlings. J. Adv. Res..

[26] Ma D., Constabel C.P. (2019). MYB Repressors as Regulators of Phenylpropanoid Metabolism in Plants. Trends Plant Sci..

[27] Liu J., Osbourn A., Ma P. (2015). MYB Transcription Factors as Regulators of Phenylpropanoid Metabolism in Plants. Mol. Plant.

[28] Korn M., Schmidpeter J., Dahl M., Voll L.M. (2015). A Genetic Screen for Pathogenicity Genes in the Hemibiotrophic Fungus *Colletotrichum higginsianum* Identifies the Plasma Membrane Proton Pump Pma2 Required for Host Penetration. PLoS ONE.

[29] Voll L.M., Zell M.B., Engelsdorf T., Saur A., Wheeler M.G., Drincovich M.F., Weber A.P., Maurino V.G. (2012). Loss of cytosolic NADP-malic enzyme 2 in *Arabidopsis thaliana* is associated with enhanced susceptibility to *Colletotrichum higginsianum*. New Phytol..

[30] Armijos C.E., Chu T.T., Connell R.J.O., Meyers B.C., Baldrich P. (2026). Stage-Specific RNA Turnover Drives Small RNA Dynamics in *Arabidopsis*-Colletotrichum Interactions. Plant Direct.

[31] Narusaka M., Abe H., Kobayashi M., Kubo Y., Narusaka Y. (2006). Comparative analysis of expression profiles of counterpart gene sets between *Brassica rapa* and *Arabidopsis thaliana* during fungal pathogen *Colletotrichum higginsianum* infection. Plant Biotechnol..

[32] Wang Y., Ye H., Ren F., Ren X., Zhu Y., Xiao Y., He J., Wang B. (2024). Comparative transcriptome analysis revealed candidate gene modules involved in salt stress response in sweet basil and overexpression of *ObWRKY16* and *ObPAL2* enhanced salt tolerance of transgenic *Arabidopsis*. Plants.

[33] Ye H., Qiao L., Guo H., Guo L., Ren F., Bai J., Wang Y. (2021). Genome-wide identification of wheat WRKY gene family reveals that TaWRKY75-A is referred to drought and salt resistances. Front. Plant Sci..

[34] Ye H., Mu J., Yang T., Shen Q., Wang Y. (2025). Integrated Transcriptome and Metabolome Analyses Reveal Candidate Genes Associate with Phenolic Compound Biosynthesis in Different Varieties of Perilla frutescens. Int. J. Mol. Sci..

[35] Zhang M., Wang Y., Cheng S., Huang W., Liu Y., Liu L. (2026). Identifying anthracnose resistance genes involving in ROS and JA accumulation. Plant Signal. Behav..

[36] Mori A., Nakagawa S., Suzuki T., Suzuki T., Gaudin V., Matsuura T., Ikeda Y., Tamura K. (2025). The importin α proteins IMPA1, IMPA2, and IMPA4 play redundant roles in suppressing autoimmunity in *Arabidopsis thaliana*. Plant J..

[37] Tang J., Xie W., Cheng Y., Fernie A.R., Zhu F. (2026). Metabolite-mediated plant immunity: From traditional defenders to artificial elicitors. J. Integr. Plant Biol..

[38] Zhang Q., Luo N., Dai X., Lin J., Ahmad B., Chen Q., Lei Y., Wen Z. (2024). Ectopic and transient expression of VvDIR4 gene in *Arabidopsis* and grapes enhances resistance to anthracnose via affecting hormone signaling pathways and lignin production. BMC Genom..

[39] Chisholm S., Coaker G., Day B., Staskawicz B. (2006). Host-microbe interactions: Shaping the evolution of the plant immune response. Cell.

[40] Iriti M., Faoro F. (2009). Chemical diversity and defence metabolism: How plants cope with pathogens and ozone pollution. Int. J. Mol. Sci..

[41] Camera S., Gouzerh G., Dhondt-Cordelier S., Hoffmann L., Fritig B., Legrand M., Heitz T. (2004). Metabolic reprogramming in plant innate immunity: The contributions of phenylpropanoid and oxylipin pathways. Immunol. Rev..

[42] Piasecka A., Jedrzejczak-Rey N., Bednarek P. (2015). Secondary metabolites in plant innate immunity: Conserved function of divergent chemicals. New Phytol..

[43] Zaynab M., Fatima M., Sharif Y., Zafar M.H., Ali H., Khan K.A. (2019). Role of primary metabolites in plant defense against pathogens. Microb. Pathog..

[44] Xie M., Zhang J., Tschaplinski T.J., Tuskan G.A., Chen J.G., Muchero W. (2018). Regulation of lignin biosynthesis and its role in growth-defense tradeoffs. Front. Plant Sci..

[45] Xia X., Wei Q., Wu H., Chen X., Xiao C., Ye Y., Liu C., Yu H., Guo Y., Sun W. (2024). *Bacillus* species are core microbiota of resistant maize cultivars that induce host metabolic defense against corn stalk rot. Microbiome.

[46] Shan X., Xia S., Peng L., Tang C., Tao S., Baig A., Zhao H. (2025). Identification of rice lncRNAs and their roles in the rice blast resistance network using transcriptome and translatome. Plants.

[47] Zhao Q., Wang H., Yin Y., Xu Y., Chen F., Dixon R.A. (2010). Syringyl lignin biosynthesis is directly regulated by a secondary cell wall master switch. Proc. Natl. Acad. Sci. USA.

[48] Xu L., Zhu L., Tu L., Liu L., Yuan D., Jin L., Long L., Zhang X. (2011). Lignin metabolism has a central role in the resistance of cotton to the wilt fungus *Verticillium dahliae* as revealed by RNA-Seq-dependent transcriptional analysis and histochemistry. J. Exp. Bot..

[49] Anjali, Kumar S., Korra T., Thakur R., Arutselvan R., Kashyap A., Nehela Y., Chaplygin V., Minkina T., Keswani C. (2023). Role of plant secondary metabolites in defence and transcriptional regulation in response to biotic stress. Plant Stress.

[50] Rao M.J., Zheng B. (2025). The role of polyphenols in abiotic stress tolerance and their antioxidant properties to scavenge reactive oxygen species and free radicals. Antioxidants.

[51] Tsuda K., Somssich I.E. (2015). Transcriptional networks in plant immunity. New Phytol..

[52] Meraj T.A., Fu J., Raza M.A., Zhu C., Shen Q., Xu D., Wang Q. (2020). Transcription factors regulate plant stress responses through mediating secondary metabolism. Genes.

[53] Rabeh K., Hnini M., Oubohssaine M. (2025). A comprehensive review of transcription factor-mediated regulation of secondary metabolites in plants under environmental stress. Stress Biol..

[54] Zhang D., Zhou H., Zhang Y., Zhao Y., Zhang Y., Feng X., Lin H. (2025). Diverse roles of MYB transcription factors in plants. J. Integr. Plant Biol..

[55] Tahmasebi A., Khahani B., Tavakol E., Afsharifar A., Shahid M.S. (2021). Microarray analysis of *Arabidopsis thaliana* exposed to single and mixed infections with cucumber mosaic virus and turnip viruses. Physiol. Mol. Biol. Plants.

[56] Medina-Puche L., Cumplido-Laso G., Amil-Ruiz F., Hoffmann T., Ring L., Rodríguez-Franco A., Caballero J.L., Schwab W., Muñoz-Blanco J., Blanco-Portales R. (2014). MYB10 plays a major role in the regulation of flavonoid/phenylpropanoid metabolism during ripening of *Fragaria × ananassa* fruits. J. Exp. Bot..

[57] Yu S., Zhang W., Zhang L., Wu D., Fu G., Yang M., Wu K., Wu Z., Deng Q., Zhu J. (2024). Negative regulation of CcPAL2 gene expression by the repressor transcription factor CcMYB4-12 modulates lignin and capsaicin biosynthesis in *Capsicum chinense* fruits. Int. J. Biol. Macromol..

[58] Tang B., Feng L., Hulin M.T., Ding P., Ma W. (2023). Cell-type-specific responses to fungal infection in plants revealed by single-cell transcriptomics. Cell Host Microbe.

[59] Pandey S.P., Somssich I.E. (2009). The role of WRKY transcription factors in plant immunity. Plant Physiol..

[60] Eulgem T., Somssich I.E. (2007). Networks of WRKY transcription factors in defense signaling. Curr. Opin. Plant Biol..

[61] Xiao S., Ming Y., Hu Q., Ye Z., Si H., Liu S., Zhang X., Wang W., Yu Y., Kong J. (2023). GhWRKY41 forms a positive feedback regulation loop and increases cotton defence response against *Verticillium dahliae* by regulating phenylpropanoid metabolism. Plant Biotechnol. J..

[62] Dong B., Liu Y., Huang G., Song A., Chen S., Jiang J., Chen F., Fang W. (2024). Plant NAC transcription factors in the battle against pathogens. BMC Plant Biol..

[63] Yuan X., Wang H., Cai J., Li D., Song F. (2019). NAC transcription factors in plant immunity. Phytopathol. Res..

[64] Aci M.M., Tsalgatidou P.C., Krommydas K., Boutsika A., Delis C., Pavli O.I., Schena L., Zambounis A. (2026). Transcriptomic insights into the biocontrol mechanism of *Trichoderma* spp. against Fusarium wilt in melon. Stress Biol..

[65] Liu Y., Su M., Zhao X., Liu M., Wu J., Wu X., Lu Z., Han Z. (2025). Combined transcriptomic and metabolomic analysis revealed the salt tolerance mechanism of *Populus talassica × Populus euphratica*. BMC Plant Biol..

[66] Shi P., Gu M. (2020). Transcriptome analysis and differential gene expression profiling of two contrasting quinoa genotypes in response to salt stress. BMC Plant Biol..

[67] Yu F., Tan Z., Fang T., Tang K., Liang K., Qiu F. (2020). A comprehensive transcriptomics analysis reveals long non-coding RNA to be involved in the key metabolic pathway in response to waterlogging stress in maize. Genes.

[68] Tian Y., Li Y., Wang K., Xia R., Lin Y., Pan G., Shi H., Zhang D., Lin H. (2024). The zinc-finger transcription factor ZFP8 negatively regulates the drought stress response in *Arabidopsis thaliana* by inhibiting the transcriptional activity of ABF2. J. Plant Physiol..

[69] Jam M., Zamani E., Tahmasebi A., Razi H., Moghadam A., Hildebrand D., Eshghi S., Alemzadeh A. (2026). Identification of common transcriptional responses to salinity of two halophytes: *Bruguiera gymnorrhiza* and *Populus euphratica*. Sci. Rep..

[70] Li J., Li Y., Du M., Zang D., Men Q., Su P., Guo S. (2024). Exogenous melatonin improves drought stress tolerance via regulating tryptophan metabolism and flavonoid biosynthesis pathways in wheat. Physiol. Plant..

[71] Gao Y., Lin Y., Liu X., García P., Liang Z. (2025). Identification of VvACO1 and transcriptional repressors VvASIL1/VvAG1 in ethylene biosynthesis of grape berries. Hortic. Plant J..

[72] Qu L.J., Zhu Y.X. (2006). Transcription factor families in *Arabidopsis*: Major progress and outstanding issues for future research. Curr. Opin. Plant Biol..

