# Supplementary material for: Transcriptomic and Metabolomic Analyses Reveal the Phenylpropanoid Metabolism Regulatory Network in Arabidopsis Response to Colletotrichum higginsianum Infection
Source: Life (Basel). 2026 Sep 9;16(9):1505. doi: 10.3390/life16091505 (PMC13608048; doi:10.3390/life16091505)
Supplement: Supplementary file 1 [file life-16-01505-s001.zip › Table S2.pdf]

Table S2. Sequencing data statistics and the result of sequence alignment between the sample sequencing data and the selected reference genome.

Sample	Total Reads	Clean reads	Clean bases	GC Content	%≥Q30	Mapped Reads	Unique Mapped Reads
Mock-R1	42,565,918	21,282,959	6,359,670,817	44.90%	96.92%	41,939,420 (98.53%)	40,672,303 (95.55%)
Mock-R2	42,239,298	21,119,649	6,313,691,171	44.92%	96.97%	41,699,352 (98.72%)	40,460,472 (95.79%)
Mock-R3	41,244,132	20,622,066	6,162,167,681	44.89%	97.01%	40,710,037 (98.71%)	39,543,993 (95.88%)
Ch-R1	43,636,192	21,818,096	6,519,235,080	44.97%	96.98%	42,965,465 (98.46%)	41,609,570 (95.36%)
Ch-R2	42,122,464	21,061,232	6,289,049,367	44.98%	96.87%	41,398,230 (98.28%)	40,133,983 (95.28%)
Ch-R3	41,048,144	20,524,072	6,132,557,135	44.96%	97.20%	40,406,234 (98.44%)	39,080,287 (95.21%)
